# On the Use of India Rubber as Adhesive Plaster

**Published:** 1855-07

**Authors:** 


### ARTICLE XIV.

### *On the Use of India Rubber as Adhesive Plaster.*

Mr. Eyre, in a communication to the London Lancet, makes the following observation respecting this subject.

If liquid India-rubber, spread upon calico, or other material, by a stiff brush, or by a knife, be used as adhesive plaster, it will be found to answer far better, in almost every case, than any other adhesive material, as it sticks firmly, is pliant, produces no irritation to the skin, and will bear lotions, or washing over it. It is also most valuable in cases where the skin requires a soft plaster of an unirritating nature for its defence, as in old persons, or others long confined to bed. In such cases, it is better to use either soft leather or the vulcanized India-rubber, made in thin sheets; the latter, from its elasticity, is often the best, as it stretches with the skin on every movement of the body. To many kinds of wounds, from operations or otherwise, strips of thin vulcanized India-rubber, spread with the liquid, will be found invaluable as elastic adhesive plasters, as they become firmly attached to the skin, and give away to all its movements. But should any wounded part require a portion of the plaster to be non-elastic, as in the case of operation for hare-lip, &c., then, in order to secure such part from being stretched, a short piece of calico, about an inch in length, should be stuck upon the middle of the elastic plaster, by which means that portion would become stationary.

If a circular piece of thin vulcanized India-rubber, about two inches in diameter, be spread with the liquid, and applied on the abdomen of an infant having umbilical hernia, and a common bandage, such as is generally used for infants, be passed lightly around the body, the protrusion will be instantly checked; and if the same plaster be again spread with the liquid and re-applied, when it comes off from time to time, no trouble will be experienced by the infant. It is not necessary to use any pad or compress.

The liquid, which is like thick treacle, and the vulcanized India-rubber, may be procured in most large towns. (They are manufactured in Manchester by Charles Mackintosh and Co.) In most cases, the thin sheet of the vulcanized India-rubber, or that sold as No. 50, will be the most suitable; in other cases, the thicker, or the No. 36, will be advisable.

The most convenient method of carrying the liquid is in one of the small, compressible bottles, used by painters, holding about an ounce, so that, on removing the screw-cap, any required quantity can be squeezed out.

The above statement will give a general idea of the subject; the materials may of course be used in a vast variety of forms.

